# Suppression of angiogenesis and tumor growth *in vitro* and *in vivo* using an anti-angiopoietin-2 single-chain antibody

**DOI:** 10.3892/etm.2014.1476

**Published:** 2014-01-07

**Authors:** ZHONG-LIN ZHANG, JI-FA ZHANG, YU-FENG YUAN, YUE-MING HE, QUAN-YAN LIU, XIAO-WEN MAO, YONG-BIAO AI, ZHI-SU LIU

**Affiliations:** 1 Hepatobiliary & Pancreatic Unit, Department of General Surgery, Zhongnan Hospital of Wuhan University, Wuhan, Hubei 430071, P.R. China; 2 Department of General Surgery, Branch Hospital in Fengxian of Shanghai No. 6 People’s Hospital, Shanghai 201406, P.R. China

**Keywords:** angiopoietin-2, phage display peptide library, single-chain antibody, neovascularization, liver tumor

## Abstract

Hepatocellular carcinomas (HCCs) are tumors with a highly developed vascular architecture. HCC cells require access to blood vessels for growth and metastasis; therefore, the inhibition of angiogenesis represents a potential therapeutic target for HCC that may reduce the mortality and morbidity from HCC. Various attempts to develop an anti-angiogenic therapy have been made in past decades; however, modest results have been achieved in clinical trials and the challenge of HCC treatment remains. Single-chain antibodies (scFv) are characterized by low molecular weight, low immunogenicity, high penetration and a short half-life, and are easy to produce on a large scale by genetic engineering. Accordingly, an scFv against a specific angiogenic regulator, such as angiopoietin (Ang), may be a promising anti-angiogenic therapy for HCC. Our previous study indicated that an imbalanced expression of angiopoietin-2 (Ang-2) vs. angiopoietin-1 (Ang-1) in HCCs contributes to initiation of neovascularization and promotes the angiogenesis and progression of HCCs. Therefore, we suggest that specific Ang-2-targeting interventions may be valuable in the treatment of HCC via remodeling the neovascular network and changing the tumor microenvironment. In this study, a prokaryotic expression vector of Ang-2 was constructed and purified human Ang-2 protein was isolated. An scFv against human Ang-2 (scFv-Ang2) was identified and purified via phage display technology, and the effects of scFv-Ang2 *in vitro* and *in vivo* on HCC in nude mice were evaluated. The results show that scFv-Ang2 inhibits vascular endothelial growth factor (VEGF) and Ang-2 induces the proliferation, migration and tubule formation of human umbilical vein endothelial cells (HUVECs) *in vitro*. In the *in vivo* assay, statistical indices, including tumor weight and volume, metastases to lungs, CD31 expression and the microvessel density (MVD) count in the scFv-Ang2-treated group of mice were significantly lower than those in the control group (P<0.05). In conclusion, the successfully generated scFv-Ang2 showed significant inhibitory effects on the angiogenesis and tumor growth of human HCC *in vitro* and *in vivo*.

## Introduction

Hepatocellular carcinoma (HCC) is one of the most malignant tumors in the tropics and the Far East, including China. It is the fourth most common cause of cancer and accounts for 53% of liver cancer mortalities worldwide (1). HCC is also a hypervascular carcinoma. Angiogenesis, a process in which endothelial cells of the pre-existing capillaries proliferate and migrate to form new vascular tips or so-called ‘vascular sprouts’ or ‘endothelial buds’ (2), plays an important role in the progression of HCC and contributes to its malignancy, invasiveness, and high rates of recurrence and metastasis (3). There is evidence suggesting that solid tumors do not grow beyond 2–3 mm^3^ in volume if vascular sprouts are blocked (2). Vascular endothelial growth factor (VEGF) is a key factor in tumor angiogenesis and high levels of VEGF have been identified to be a determining factor in the HCC grade, clinically correlating with low rates of overall survival (4–6). As a result, the development of biomedical research on vascular biology is paramount to the foundation of genetic engineering and proteomics technology that may provide a new and effective angiogenesis-targeting therapy for HCC.

HCCs are tumors with a highly developed vascular architecture; HCC cells require access to blood vessels for growth and metastasis. Therefore, the inhibition of angiogenesis represents a potential therapeutic target for HCC (7). In recent years, research in this area has been highly active and numerous anti-angiogenic agents have been developed for specific clinical indications (8). However, anti-angiogenic drugs have produced modest results in clinical trials despite significant therapeutic effects demonstrated *in vitro* or *in vivo* (9,10). Overall, these drugs have yet to contribute to long-term survival benefits (11). Single-chain antibodies (scFv) are characterized as highly penetrating proteins with low molecular weight, low immunogenicity and a short half-life. The large-scale production of scFv is easy to implement by genetic engineering (12). Therefore, scFv as direct therapeutic agents or as carriers of cytotoxic agents for specific targeted therapies are promising for clinical applications, including HCC therapy.

Tumoral angiogenesis is a complex process closely regulated by numerous angiogenic factors, among which angiopoietin and VEGF are the two most significant. VEGF is the most potent angiogenic factor that promotes endothelial proliferation and increases vascular permeability by binding to its specific receptors in endothelial cells, including Flt-1, KDR/Flk-1 and Flt-4 (13). Angiopoietin-2 (Ang-2) has been found with abnormally high expression levels in numerous solid tumors, including gastric, ovarian, colorectal and breast cancers (14–17). Ang-2 is thus considered one of the most important tumoral angiogenesis promoters. Animal models and *in vitro* experiments have shown that Ang-2 and its receptor Tie2, in association with VEGF, constitute a system that regulates vascular quiescence and endothelial plasticity, through which a balanced state of vascular maturity and development of complex vascular networks are achieved (13). Ang-2 in the presence of VEGF is important for the initiation of angiogenesis and vascular sprouting in tumors (18). It has been reported that VEGF and the angiopoietin/Tie2 system play a key role in the transformation of normal lung to non-small cell lung carcinoma (19). Our previous study (20) indicated that expression of Ang-2 relative to that of angiopoietin-1 (Ang-1), through the Tie2 receptor in the presence of VEGF, plays a critical role in initiating early neovascularization and induces the transformation of noncancerous liver to HCC. Subsequently, constant immature neovascularization in HCC further promotes angiogenesis and tumor progression. Therefore, we suggest that Ang-2-targeting therapies may be valuable in the treatment of HCC by intervening in the remodeling of neovascular networks and changing the microenvironment of the tumor.

In this study, a prokaryotic expression vector of Ang-2 was constructed and purified human Ang-2 protein was isolated. A single-chain antibody against human Ang-2 (scFv-Ang2) was identified, which was purified with phage display technology. Finally, the effects of scFv-Ang2 *in vitro* and *in vivo* on HCC in nude mice were evaluated.

## Materials and methods

### Reagents

The following reagents were obtained: pET32c vector system from Novogen (Madison, WI, USA); plasmid pCANTAB5E, *Escherichia coli* TG1 and *Escherichia coli* BL21, M13K07 helper phage, mouse anti-M13 antibody and mouse anti-E tag antibody from Pharmacia Biotech (Piscataway, NJ, USA); pfuDNA polymerase from Stratagene (Santa Clara, CA, USA), restriction endonuclease *Hin*dIII, *Nco*I and T4 DNA Ligase from New England Biolabs (Ipswich, MA, USA); low melting point agarose from Promega (Madison, WI, USA); isopropyl β-D-1-thiogalactopyranoside (IPTG), total RNA kit and Moloney murine leukemia virus (M-MLV) reverse transcriptase from Takara (Shiga, Japan); protein marker and FastDigest restriction enzymes from MBI Fermentas Inc. (Burlington, ON, Canada); Rapid Gel purification kit and PureLink™ HiPure plasmid DNA purification kit from Invitrogen (Carlsbad, CA, USA); enterokinase and 3-(4,5-dimethylthiazol-2-yl)-2,5-diphenyltetrazolium bromide (MTT) from Sigma (St. Louis, MO, USA); M199, fetal bovine serum (FBS) and trypsin-ethylenediaminetetraacetic acid (EDTA) from Hyclone (Logan, UT, USA); Dulbecco’s modified Eagle’s medium (DMEM) from PAA Laboratories GmbH (Linz, Austria); endothelial cell growth supplement ECGs) from Millipore (Temecula, CA, USA); collagenase from Worthington Biochemica (Lakewood, NJ, USA); mouse anti-human CD31 monoclonal antibody from Santa Cruz Biotechnology, Inc., (Santa Cruz, CA, USA); EDTA, penicillin and streptomycin from Invitrogen; and dimethyl sulfoxide (DMSO) and Transwell chambers with 8-μm pore filters from Corning Incorporated (Corning, NY, USA). Matrigel was obtained from BD Biosciences (Franklin Lakes, NJ, USA). Recombinant human VEGF was obtained from R&D Systems (Minneapolis, MN, USA).

### Cell culture

Human umbilical vein endothelial cells (HUVECs) were isolated from human umbilical cords using collagenase and were cultured in M199 medium containing inactivated 20% FBS and 25% ECGs. The cells were maintained in a humidified 5% CO_2_ atmosphere at 37°C. Cells were divided at a ratio of 1:2 once they reached 70–90% confluence. The cells were grown into a monolayer within 2–3 days and continually cultured for 2–3 passages prior to experimentation. MHCC97 (human liver cancer cell line) cells were obtained from the Liver Cancer Institute of Fudan University and incubated in high glucose DMEM with 10% FBS at 37°C in a humidified atmosphere of 95% air and 5% CO_2_. Experiments were conducted on cultures that had gone through 2–3 passages. Written approval for human umbilical vein endothelial cell derivation, culture, and experimental use was obtained from the Ethics Committee, Zhongnan Hospital of Wuhan University.

### Human HCC model in nude mice

Male athymic BALB/c nu/nu mice, 4–6 weeks old, were obtained from The Experimental Animal Center of Wuhan University (Animal Biosafety Level-III Laboratory; Wuhan, China) and maintained in specific pathogen-free (SPF) conditions. The study protocol for the use of mice was approved by The Wuhan Medical Experimental Animal Care Commission (Wuhan, China).

The metastatic model of human HCC in nude mice was constructed via orthotopic implantation of histologically intact metastatic tumor tissue. Briefly, 5×10^6^ (0.2 ml) MHCC97 cells were injected subcutaneously into the nude mice. When the subcutaneous tumor reached ~1.5 cm in diameter, the mice were sacrificed and small pieces of tumor tissue, ~1 mm^3^ in volume, were implanted into the livers of new recipient mice, which were maintained in standard facilities.

### Construction of human Ang-2 prokaryotic expression vector

#### Gene amplification

The total RNA was isolated from HUVECs with RNAiso reagent (Takara) following the manufacturer’s instructions and was quantified by absorbance analysis at 260 nm. cDNA was generated by reverse transcription using an oligo(dT) primer and RNA PCR kit (Takara). According to the original sequence in Gene Bank (accession number: AF004327), the method of Maisonpierre *et al* (21) was used to synthesize the Ang-2 gene. *Nco*I and *Hin*dIII restriction sites were inserted into the 5′ and 3′ ends of primers, respectively. Quantitative PCR (qPCR) was performed using a GeneAmp PCR system 2400 (Perkin Elmer, Waltham, MA, USA). After equal amounts of cDNA and specific primers were added to the master mix, initial denaturation at 94°C for 30 sec, followed by 35 cycles of denaturation at 94°C for 3 min, annealing at 59°C for 60 sec and extension at 72°C was conducted. PCR products (5 μl) were visualized by electrophoresis on 1% agarose gel, stained with ethidium bromide and purified using a Rapid Gel purification kit, and were also sequenced by Davis Sequencing (Davis, CA, USA). The sequence was compared with the matching portion of that submitted to the NCBI.

The purified PCR products and empty plasmid pET32c were digested by *Nco*I and *Hin*dIII restriction enzymes, harvested by electrophoresis on 1% agarose gel and purified using a Rapid Gel purification kit. Following ligation to pET32c by incubation with T4DNA ligase at 16°C overnight, the vector was then used to transform competent *E. coli* BL21 by spreading on agar plates with ampicillin at 37°C overnight. A single colony of *E. coli* BL21 containing the recombinant plasmid pET32c-Ang-2 was inoculated and selection was performed in LB-Amp medium. The alkaline lysis method was used for plasmid extraction in the mini-preparation scale and the PureLink™ HiPure plasmid DNA purification kit was used for mini-preparation DNA extraction. The accuracy of cloning was confirmed using restriction enzyme mapping and sequencing. The plasmids were extracted using PureLink™ HiPure plasmid DNA purification kit and were digested with the *Nco*I and *Hin*dIII restriction endonuclease enzymes. For each reaction 8 μl purified plasmid, 2 μl buffer, 2 μl enzyme (1 μl of each in double digestion) and 8 μl ddH_2_O were added. The digestion was performed for 5 min in 37°C with FastDigest restriction enzymes (New England Biolabs). Final confirmations of positive clones were performed by PCR and DNA sequencing.

#### Expression and purification of target fusion protein

To examine whether pET32c-Ang-2 overexpressed Ang-2, pET32c-Ang-2 was transfected into the *E. coli* strain BL21 (DE3) that encodes a chromosomal T7 RNA polymerase under the control of a tac promoter. A single colony was then inoculated on LB medium and grown at 37°C overnight with constant agitation. A 200-μl sample of the overnight products was collected and added to 3 ml LB-Amp medium, which was cultured on a shaking plate at 37°C until the sample reached an OD_600_ of 0.6. IPTG was added at a concentration of 1 mmol/l to induce pET32c-Ang-2-BL21 (DE3) expression. Bacterial liquid was centrifuged at 3,099 × g for 15 min at 4°C and whole protein was collected for SDS-PAGE gel analysis, according to Novagen’s pET System Manual. Protein was recovered by electroelution and dialysis methods, and its concentration was determined by Lowry protein assay.

Fusion protein of Ang-2, which was expressed downstream of restriction enzyme site of enterokinase, was digested by enterokinase at 25°C in a water bath so that pure Ang-2 protein was obtained. Different concentrations of enterokinase with different durations of digestion were tested to determine digestion efficiency. Large quantities of target protein were prepared and concentrated for later use after the optimal preparation conditions were determined.

### Preparation of scFv against Ang-2 by phage display peptide panning

A non-immunized mouse phage display antibody library was constructed and screened as previously described (22). To display scFv as a fusion protein with E tag and M13 p3 (there is an amber stop codon between E tag and fd g3 in pCANTAB5E), scFv-Ang2 DNAs were ligated into the phagemid vector pCANTAB5E. The ligated products were used to transform competent *E. coli* TG1 cells: TG1 is an amber suppressor strain (supE). The panning process was performed according to the method of Carlos *et al* (23). Three rounds of panning and enrichment were performed, and the affinities of recombinant phage antibodies from pooled or individual colonies following each round of selection were tested by enzyme-linked immunosorbent assay (ELISA). Finally, screened scFv-Ang2 was identified by SDS-PAGE and DNA sequencing.

### In vitro experiments

#### Treatment groups

The HUVECs were planted in culture plates with 3 wells for each group. Five groups were used: Group 1 was set as the control group with no other external factors added, with the exception of ECGs for maintaining growth in the M199 medium; group 2 contained VEGF (5 μg/l); group 3 contained Ang-2 (10 μg/l); group 4 contained VEGF (5 μg/l) and Ang-2 (10 μg/l); and group 5 contained VEGF (5 μg/l), Ang-2 (10 μg/l) and scFv-Ang2 (1×10^11^, 2×10^11^ and 4×10^11^ pfu/ml, respectively).

#### MTT assay

The cell viability was determined using the MTT assay. Briefly, HUVECs (2×10^4^/well) were seeded onto 96-well plates and incubated for 24 h for synchronization. After adherence was attained, the various treatments were added, with 3 wells for each group. Following incubation for 24 h, 20 μl MTT reagent (5 mg/ml) was added to each well and the cells were incubated for 4 h. The formazan precipitate was dissolved in 150 μl DMSO and the absorbance value was measured with a microplate reader (ELx808; BioTek, Winooski, VT, USA) at a wavelength of 490 nm.

#### Cell migration assay

Transwell plates of 8-μm pore size were used to evaluate cell migration ability. The polycarbonate filter of the inner chamber was coated with 1% gelatin and equilibrated with serum-free DMEM for 1 h. HUVECs were harvested and resuspended in DMEM medium supplemented with 20% FBS. The prepared cell suspension (100 μl; 4×10^5^ cells/ml) was added to the inner chamber and the outer chamber was filled with 600 μl DMEM containing FBS. The cells were fixed with 90% methanol for 10 min following incubation at 37°C for 6 h. Following the removal of cells on the upper side of the membrane using a cotton swab, the cells were stained with crystal violet for 10 min and washed with PBS. The cells were observed and counted using an inverted microscope (CKX41; Olympus, Tokyo, Japan) at ×400 magnification and the average value of three fields was calculated.

#### Tubule formation assay

Growth factor-free Matrigel maintained at 0°C was added to a 96-well microplate and maintained at 37°C for 1 h. HUVECs at 50 μl/well (2×10^4^/ml) were seeded onto an extracellular matrix with different factors as previously grouped. After culturing for 18 h, three random ×100 fields were photographed under a phase-contrast microscope (CX41; Olympus) for each well, and the tubules were counted, averaged and compared.

### In vivo anti-tumor experiment

#### Animal grouping

The animals were grouped as follows: 24 nude mice were randomly divided into therapy and control groups with twelve animals in each group. scFv-Ang2 (4×10^11^ pfu; 1 ml) was intraperitoneally administered once a day in the therapy group and saline was administered at the same volume and frequency in the control group. The injection course started from the second day of inoculation and continued for the following 3 weeks. The body weight of the mice was recorded once a week.

#### Parameters observed

On day 21, the mice were sacrificed. Tumors were weighed and the longest (a) and the smallest (b) diameters were measured by slide gauge under an operating microscope. Tumor volume was calculated as follows: V=a.b^2^/2. The liver tissues were carefully anatomized and visible metastases were counted. Paraffin blocks of 10% buffered formalin-fixed samples of lungs were prepared. Each lung sample was consecutively cut into 10 slices every 30 μm. Serial sections were cut at 5 μm and stained with hematoxylin and eosin to determine the presence of lung metastases. Tumor tissue was embedded in a paraffin block for advanced immunohistochemistry analysis of CD31.

#### Immunohistochemical assessment of vessel density

Paraffin-embedded tumor tissues were sectioned (4 μm), the slides were deparaffinized and washed with Tris-buffered saline (TBS), and the slices were incubated with 10% normal goat serum (Zhongshan Bio., Guangzhou, China). The sections were then incubated with appropriately diluted (1:10) rat-anti-mouse CD31 monoclonal antibody for 24 h at 4°C. The primary antibody was removed and, after washing the sections with TBS, peroxidase-labeled goat-anti-mouse IgG (Zhongshan Bio.) was added. Finally the slices were stained with hematoxylin, and washed with distilled water. Quantification of blood vessels was carried out as previously described (24). A brown-stained endothelial cell cluster distinct from adjacent microvessels, tumor cells or other stromal cells was considered as a single countable microvessel. The most vascular areas of tumors were identified on a low-power field (x100) and vessels were countered in five high-power fields (x200). The data are expressed as mean ± standard deviation (SD) for five high-power fields.

### Statistical analysis

The data were analyzed for significance with an unpaired Student’s t-test and ANOVA test. Statistical software SPSS version 13.0 (SPSS, Inc., Chicago, IL, USA) was used in the analysis. P<0.05 was considered to indicate a statistically significant result.

## Results

### Construction of human Ang-2 prokaryotic expression vector

**Figure 1:**
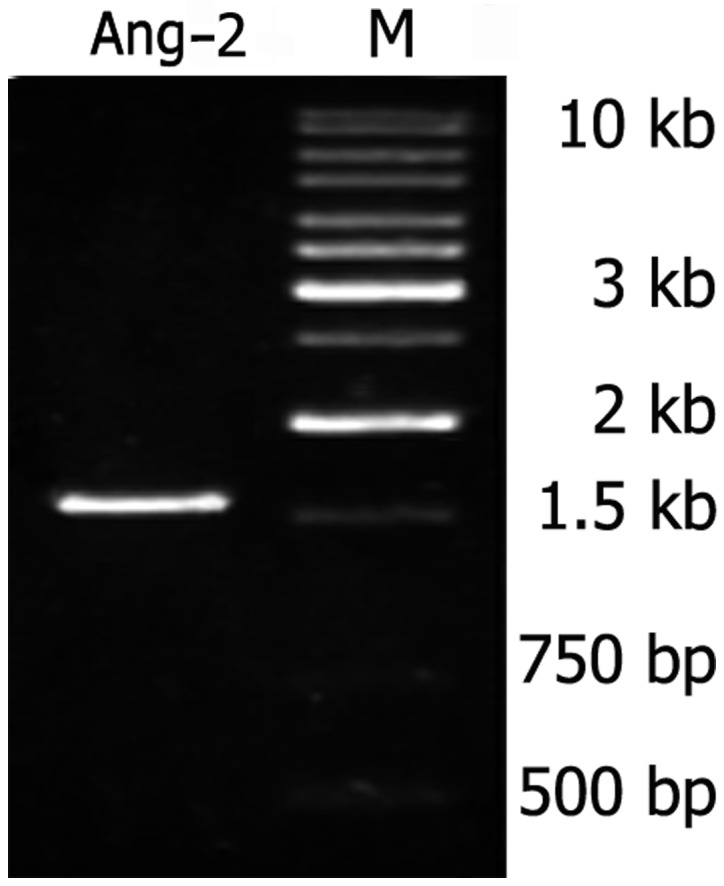
Presence of Ang-2 RNA by qPCR. Lane M, marker, GeneRuler 1 kb DNA Ladder. The band of Ang-2 was observed at the predicted location on the gel. Ang-2, angiopoietin-2; qPCR, quantitative PCR.

As shown in Fig. 1, the Ang-2 gene produced a specific band at approximately 1.5 kb in the agarose gels when it was amplified from the HUVEC cDNA library. The obtained PCR products had identical cDNA sequences to the gene bank sequences (data not shown).

**Figure 2:**
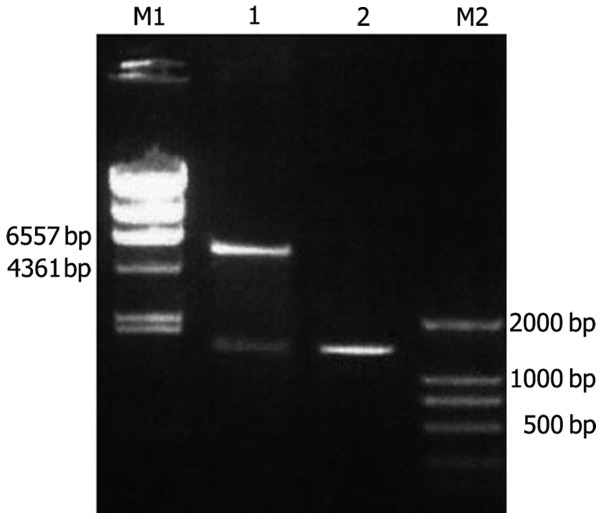
Electrophoresis of recombinant plasmid DNA. Agarose gel (0.8%) showing the results of PCR and the digested clone fragments. Lanes: M1, λDNA/*Hin*dIII DNA marker; 1, digested clone fragments of pET32c-Ang-2 plasmid; 2, Ang-2 gene; M2, DL2000 DNA marker. Ang-2, angiopoietin-2.

The amplified Ang-2 gene was inserted into the pET32c vector and was consequently transformed into the *Escherichia coli* strain BL21, as confirmed by sequencing and digestion with *Nco*I and *Hin*dIII (Fig. 2).

**Figure 3:**
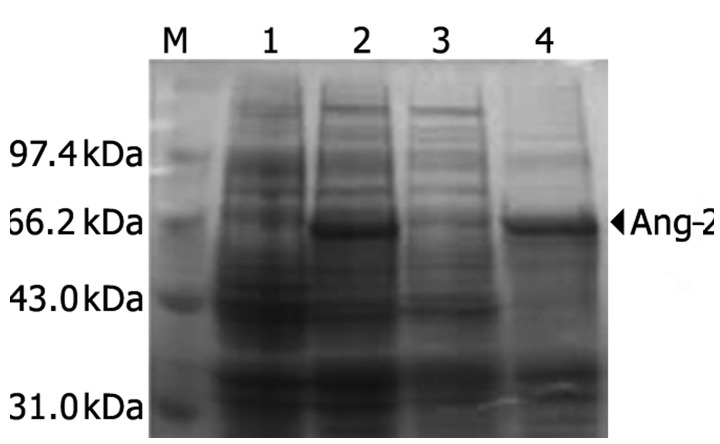
SDS-PAGE analysis for protein expression. Lanes: M, marker for low molecular weight proteins; 1 and 2, total bacteria before and after induction, respectively; 3 and 4, supernatant and precipitation after induction, respectively.

Recombinant plasmid pET32c-Ang-2 was transformed into *Escherichia coli* BL21 and was strongly expressed following IPTG induction at 15°C. The 12% SDS-PAGE electrophoresis result (Fig. 3) indicated that Ang-2 protein was solubly expressed in the pET32c system and was mainly observed in the supernatant. The human Ang-2 consists of 489 amino acids and predicts a molecular weight of 56.9 kDa. Together with glycosylation of the N terminal amino acid, Ang-2 migrates approximately to the 60–70 kDa band in SDS-PAGE under reducing conditions. After optimizing the conditions, Ang-2 protein was prepared on a large scale and stored for subsequent study.

**Figure 4:**
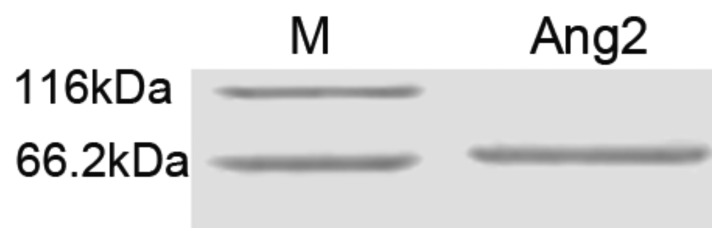
SDS-PAGE of purified Ang-2 protein. Molecular weight is ~70 kDa. Lanes: M, marker; Ang-2, angiopoietin-2 protein.

The complete Ang-2 protein was obtained by enterokinase digestion at the optimal conditions of 25°C in a water bath for 4 h. SDS-PAGE of the purified protein products is shown in Fig. 4. The protein was stored at -70°C following concentration.

### Generation of scFv against Ang-2

#### Phage display antibody screening

Purified Ang-2 protein was used to screen antibodies from a phage display library. Three rounds of selection against Ang-2 showed specific enrichment of phage antibodies. One clone was obtained with binding activity to Ang-2, as shown in Table I.

#### Results of ELISA assays

**Figure 5:**
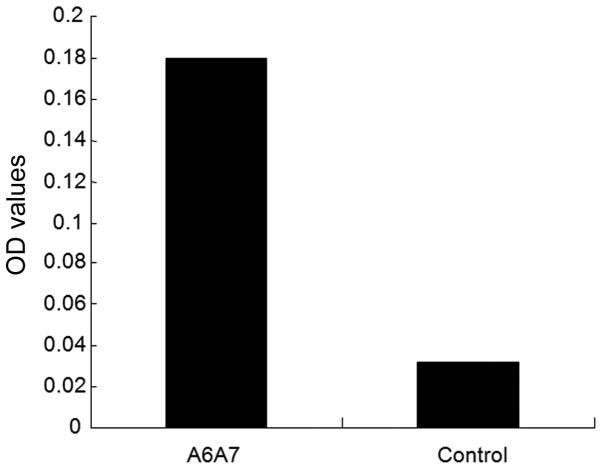
Comparison of OD values between positive and negative clones. A6A7 represents positive clone culture.

After 3 rounds of panning, 96 individual clones of phage-scFv-Ang2 were randomly selected and cultured in a 96-well plate, 50 μl centrifuged bacteria liquid was obtained and tested by cell ELISA. As shown in Fig. 5, positive phage clone A6A7 showed a significantly higher OD value than the negative (control) group (P<0.05), which demonstrated the high binding affinity of the phage-scFv-Ang2 clone for Ang-2 protein.

#### Electrophoresis of the scFv gene

**Figure 6:**
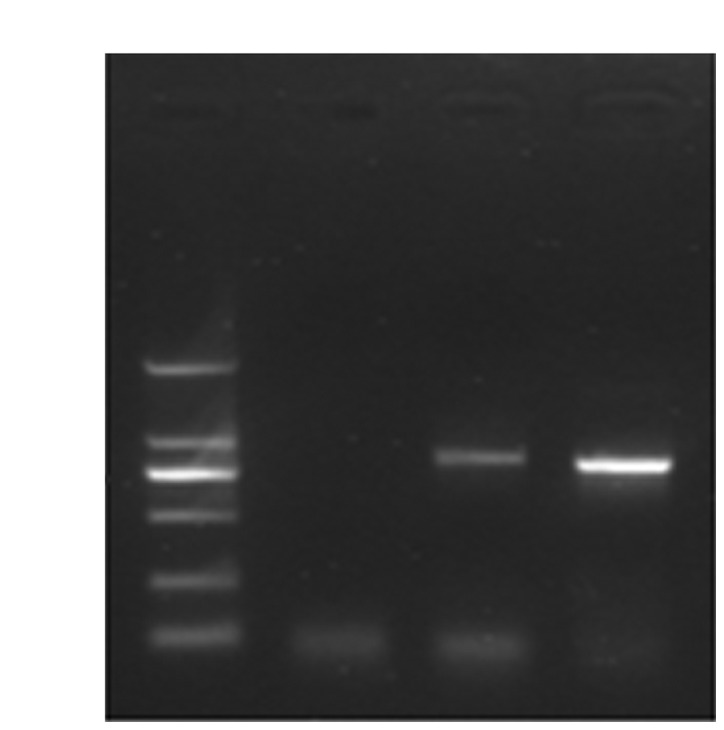
Electrophoresis of scFv-Ang2 gene following PCR. M, DL2000 marker; 1, negative control; 2 and 3, products of PCR for plasmid; 2, A6A7; 3, positive control. scFv, single-chain antibody; Ang-2, angiopoietin-2.

The positive clone was screened and amplified by PCR. Electrophoresis of the products in agarose gel (15 g/l) indicated a 750-bp specific scFv-Ang2 fragment (Fig. 6).

#### DNA sequencing for scFv-Ang2

The result of DNA sequencing (data not shown) indicated that the obtained scFv gene had an open reading frame (ORF) of 912 encoding base pairs, which suggests a target protein with a molecular weight of ~33.7 kDa.

#### Identification of scFv by SDS-PAGE

**Figure 7:**
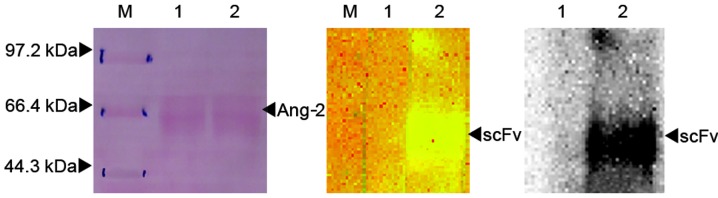
Immunoblot analysis of scFv displayed on phage against Ang-2. Left: Ponceau S stained nitrocellulose membrane after SDS-PAGE. Right: image of immunoblot detected by chemiluminescence. Middle: overlay image from chemiluminescence and white light showing molecular weight bands. Ang-2 was run on SDS-PAGE and transferred to a nitrocellulose membrane. M, protein marker; Lane 1, negative control without scFv displayed on phage use as used as primary antibody; Lane 2, result of scFv displayed on phage against angiopoietin-2. Ang-2, angiopoietin-2; scFV, single-chain antibody.

Fig. 7 shows that the scFv-Ang2 phage specifically recognized Ang-2 protein.

### Effects of scFv-Ang2 on angiogenesis and growth of HCC

#### Effects on proliferation of HUVECs

An MTT assay was used to determine the absorbance value of HUVECs in different groups treated with various stimulatory factors. The results obtained and comparison between groups are shown in Table II. The proliferation capacity of HUVECs significantly increased when VEGF only was added, which showed a VEGF-dependent enhancement. No effect was observed when Ang-2 protein only was added. In the VEGF and Ang-2 combination group, the HUVECs showed the highest proliferation ability with a significant increase, however, such ability was significantly inhibited when scFv-Ang2 was added (VEGF + Ang-2 + scFv-Ang2), which also showed a marked concentration-dependent inhibitory effect.

#### Effects on migration of HUVECs

As shown in Table II, VEGF and Ang-2 promoted HUVEC migration compared with that in the control group. A combination of the two showed the most potent migration-promoting effect. Concentration-depend inhibition on cell migration was observed when scFV-Ang-2 was added.

#### Effects on tubule formation of HUVECs

**Figure 8:**
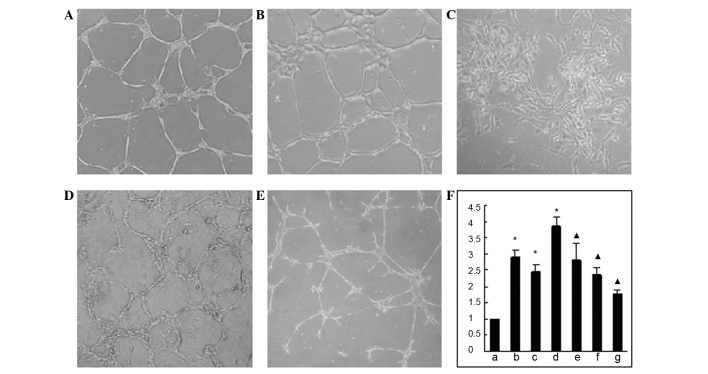
Tubule formation of HUVECs and column graph of tubule formation index in different groups. (A) Control group; (B) Ang-2 group; (C) VEGF group; (D) VEGF+Ang-2 group; (E) VEGF+Ang-2+scFv-Ang2 group; (F) column graph of tubule formation index in different groups, in which (a) represents control group data, (b) Ang-2 group, (c) VEGF group, (d) VEGF+Ang-2 group, (e–g) represents VEGF+Ang-2+scFv-Ang2 groups with scFv-Ang2 concentrations of (e) 1×10^11^ pfu/ml; (f) 2×10^11^ pfu/ml; (g) 4×10^11^ pfu/ml. ^▲^P<0.05, compared with the VEGF+Ang-2 group; ^*^P<0.05, compared with the control group. HUVECs, human umbilical vein endothelial cells; VEGF, vascular endothelial growth factor; Ang-2, angiopoietin2; scFv, single-chain antibody.

The Matrigel tubule formation assay results showed that the group treated with a combination of Ang-2 and VEGF displayed the most increased endothelial cell migration. The HUVECs were stretched to an increased size and tended towards a tubular structure formation among endothelial cells and the number of tubules formed was significantly higher than that of the control group (P<0.05). However, when scFv-Ang2 was added, these effects were significantly inhibited, and it was observed that the tubules formed in the VEGF+Ang-2+scFv group were smaller in number and had a poorer integrity of lumenal structure than those of the control group (P<0.05), as shown in Fig. 8. The tubule formation index was calculated according to the relative ratio of the tubule number in the intervention group to that in control group; the comparison data are shown in Fig. 8F.

#### Tumor growth in mice

Lumps in the stomach and skin invasion were observed in the fifth week when the mice were sacrificed. The changes in tumor weight and volume in the treatment groups were significantly smaller than those in the control group, and were 0.9301±0.2842 vs. 1.4483±0.3633 g and 0.5981±0.3925 vs. 1.1806±0.3188 cm^3^, respectively.

#### Effects on HCC metastasis

**Figure 9:**
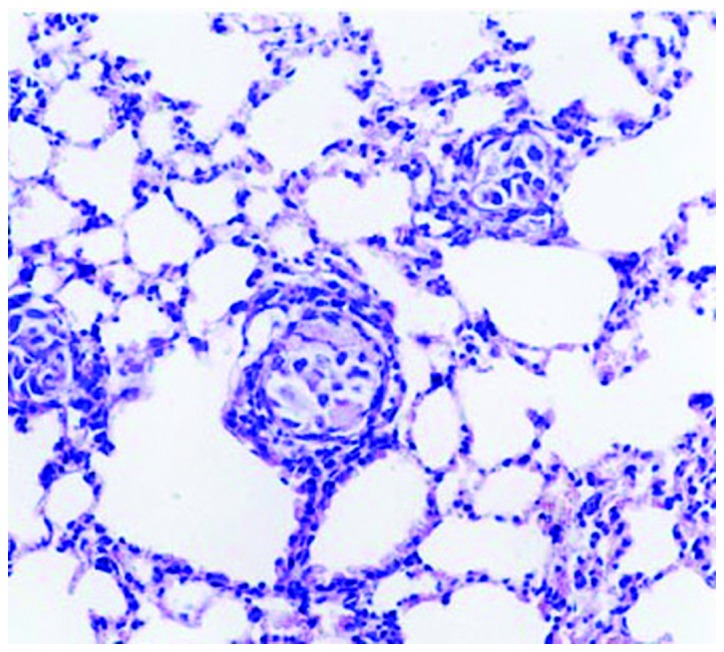
Metastases of hepatocellular carcinoma in lungs. Magnification, ×200. Staining, hematoxylin and eosin.

**Figure 10:**
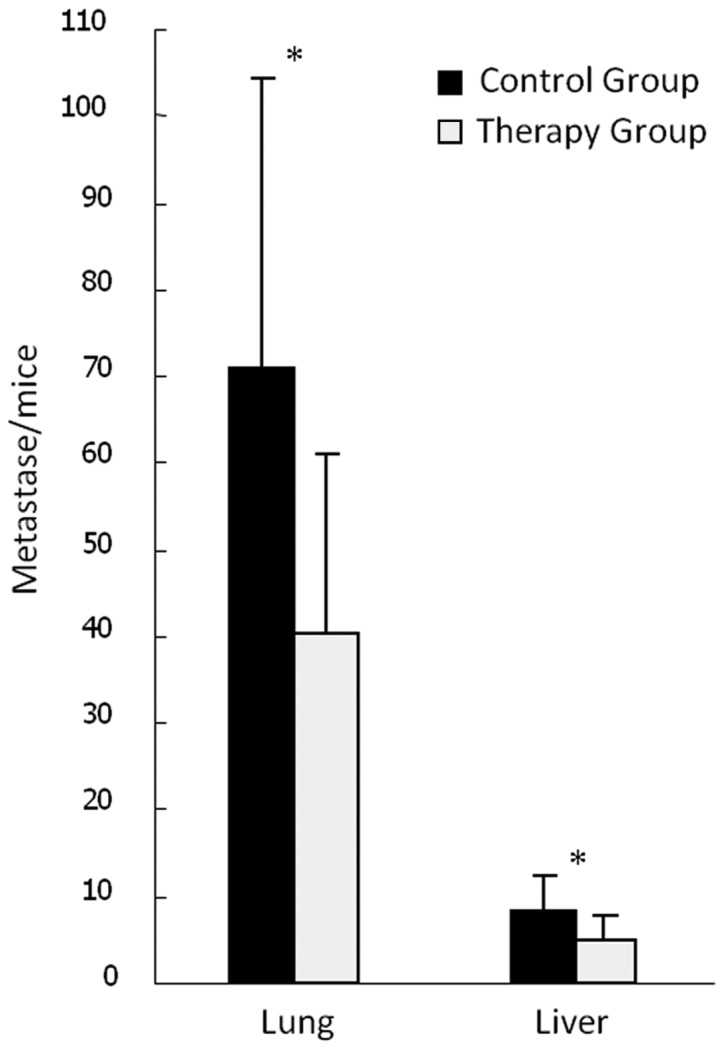
Metastases of hepatocellular carcinoma in the lungs and the liver. ^*^P<0.05, compared with the control. The liver metastases were recorded in a gross manner by examining each lobe of the liver and counting macroscopic tumors on the surface. For the lung, metastases were counted under a microscope by observing consecutive paraffin slices of lung.

Visible metastases were observed when seven lobes of liver were carefully anatomized and the number of visible metastases was recorded. Gross pathological examination of the lungs identified scattered hemorrhagic spots, which were confirmed by histopathology to be metastases (Fig. 9). The metastatic rate in the liver and lungs in the treatment and control groups was 100%, but the numbers of metastases in both liver and lungs in the therapy group were significantly reduced compared with those in the control group (P<0.05, Fig. 10); the numbers of metastases were 4.75±3.10 vs. 8.25±4.00 in the liver and 40.25±20.79 vs. 70.75±33.60 in the lungs, respectively.

#### Expression of CD31

**Figure 11:**
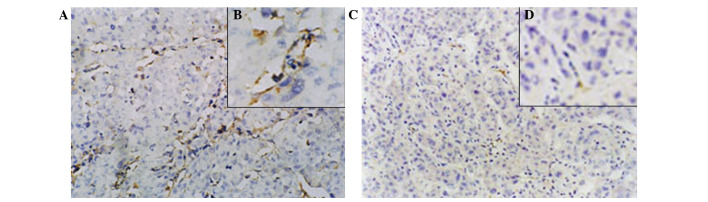
Immunohistochemical expression of CD31 in tumor tissues. (A and B) experimental groups; (C and D) control group. (A and C) magnification, ×200; (B and D) magnification, ×400.

The expression of CD31 in the experimental groups was poor or even absent, whereas it was strong in the control group. The newborn endothelial cells were stained brown or yellow and sinusoidally distributed in the capillary walls of portal area and fiber interval of the liver tissue (Fig. 11). Microvessel counting revealed that microvessel density (MVD) in the control group was 70.00±16.27 per high-power field (x200), whereas it was 21.08±6.23 in the therapy group (P<0.05).

## Discussion

Among the various angiogenic regulators, the vascular endothelial cell-specific receptor tyrosine kinase (RTK) family plays the most important role in the regulation of the endothelial cell function (25,26). Within the family, the function of the angiopoietin/Tie2 system within the process of neovascularization has gained attention in recent studies (27). The angiopoietin family, including Ang-1, -2, -3 and -4, have been isolated and identified as a group of ligands of the tyrosine kinase Tie2 receptor (28). Ang-1 and Ang-2 have been demonstrated as the most potent regulators for neovascularization (29) and are the activator and antagonist of the Tie2 receptor, respectively, where binding of Ang-1 causes autophosphorylation of Tie2 whereas Ang-2 binding suppresses the autophosphorylation (29,30). It was reported that normal regulation of tyrosine kinase Tie2 is required for normal vascular development, by regulating vascular remodeling and maturation (31). Ang-1 contributes to the maintenance and stabilization of maturation vessels by promoting interaction between endothelial cells and support cells, such as pericytes. Knockout mice deficient in angiopoietin-1 develop severe vascular defects and die *in utero*, in a similar manner to Tie2 deficiency in mice (32). Ang-2 acts as an alternative ligand for Tie2 and binds to Tie2 with similar affinity, but competitively antagonizes the effects of Ang-1 by inhibiting Tie2 phosphorylation and activation. Functionally, transgenic mice overexpressing Ang-2 show even more severe vascular defects than the Ang-1 or Tie2 deficient mice (12). In the presence of VEGF, vessel destabilization caused by Ang-2 has been hypothesized to induce an angiogenic response, whereas in the absence of VEGF, Ang-2 leads to vessel regression (21,33). Previous studies (34,35) suggested that highly expressed Ang-2 in tissues contributed to tumoral angiogenesis and correlated with tumor growth and poor prognosis. It has also been reported (36) that Ang-2 may be one of the most important risk factors for the postoperative recurrence of HCC following hepatic resection. In addition, patients with high levels of preoperative Ang-2 mRNA are more likely to have a poorer survival rate than those with low preoperative Ang-2 mRNA levels following surgery. Our previous study confirmed that overexpression of Ang-2 and VEGF was a key promoter of early neovascularization and consequent carcinogenesis, and contributed greatly to the invasion and metastasis of HCC, considered as a typical hypervascular carcinoma. Angiogenesis thus contributes to its poor prognosis (20). The promotory effect of Ang-2 on angiogenesis is considered a potential target for vascular proliferative diseases, such as HCC.

Ang-2-targeting intervention through the immunological technique of antigen-antibody binding was one of the most important molecular biological methods. Angiogenic gene therapy based on a molecular level has been attempted in recent years; however, due to the complicated process of exogenous genes entering the body and realizing biologically active protein expression through transcription and translation, the significant efficacy of *in vitro* experiments was not demonstrated *in vivo* in the majority of studies, and therapeutic effects in clinical trials remain difficult to attain (37). Modern phage display peptide library screening technology allows the use of proteomic methods to achieve screening and preparation of a high-affinity scFv against a specific target. Smith first described phage display technology based on the ability to express foreign (poly)peptides as fusions to capsid proteins on the surface of bacteriophages in 1985 (38). Surface display is achieved by inserting a peptide-encoding gene into the gene for a capsid structural protein. Billions of pooled peptides presented on phage particles form a phage-displayed peptide library, and in contrast to regular synthetic small molecule libraries, as many as 1,010 different peptides may be screened simultaneously for the desired activity (39,40). In the present assay, target phage with the specific protein that binds with phage peptide sequences through a ligand-receptor type binding mode was panned out from the random peptide phage library and further amplification was realized by transfecting *Escherichia coli*. The target protein (scFv) was identified and purified, and its structure and functions were analyzed. The scFv may be expressed in *E. coli* as a single-chain molecule in which the heavy chain (VH) and light chain (VL) domains of the antibody are joined by a flexible polypeptide linker (41). Over the past two decades, phage display has been widely used in numerous scientific fields including drug discovery/design, such as screening for receptor agonists and antagonists, drug target validation, development of vaccines, *in vitro* selection of new antibodies, antibody fragments and antibody surrogates as randomized fragments on diverse scaffold proteins, and discovery of agents for the targeted delivery of drugs and gene therapy.

Compared with whole antibody molecules, the scFv contains the variable region of antibody and the complete antigen-binding site, but does not contain the fragment crystallizable region (Fc region), which significantly increases the specificity of the antigen-antibody binding. In addition, its low molecular weight, low immunogenicity, high penetration and ease of production on a large scale (12) makes it a particularly promising therapeutic agent.

The construction of a phage display peptide library is a complex task. In order to avoid this in preliminary experiments, a non-immunized mouse phage display antibody library built by the Institute of Hydrobiology, Chinese Academy of Sciences (Wuhan, China) with a storage capacity of 10^9^ was used (22). Therefore, the complex process of repeatedly constructing an antibody library to target various antigens was avoided, the majority of antibody genes were collected in the library and the diversity of antibodies was significantly increased. It was easy to access a variety of protein antigens of specific scFv from the antibody library (42). Biologically active Ang-2 protein was used to reduce interferences during the panning process. Screening and the identification of scFv-Ang2, which contained 303 amino acids with a molecular weight of ~33.7 kDa was successfully conducted. The specificity of the intact antibody molecule was demonstrated in the present study. The obtained scFv-Ang2 was first used *in vitro* in primary cultured HUVECs to observe its effects on vessel formation. The results showed that scFv-Ang2 alone did not promote the proliferation of HUVECs, but was able to promote endothelial cell migration and tubule formation. When scFv-Ang2 was combined with VEGF the angiogenic ability of the latter was significantly enhanced. This confirmed that Ang-2 plays an important role in the process of angiogenic regulation and may be valuable as a target for intervention. As Ang-2 itself does not promote HUVEC proliferation, its function of regulating neovascularization may be dependent on cooperative action with other angiogenic factors, such as VEGF. Interactions between Ang-2 and VEGF have also been confirmed in systems other than tumors, such as a corneal neovascularization model (43). Another important angiogenic factor angiotensin II (ANGII) has been shown to cause increased expression of Ang-2 (44,45). However, the mechanism of how these factors increase Ang-2 expression has not been investigated. We speculate that Ang-2-mediated downstream signaling pathways may be the common mechanism of action for numerous angiogenic factors. In vascular proliferative disorders, such as cancer and diabetic retinopathy, the blockade of Ang-2 may lead to anti-angiogenesis from Ang-2 inhibition and the inhibition of other angiogenic factors whose activities are mediated by Ang-2 (46). When scFv-Ang2 was added to cultured endothelial cells containing Ang-2, the promotion of endothelial migration and tubule formation was significantly reduced, which indicates that scFv-Ang2 was effective *in vitro* in blocking Ang-2 and inhibiting its angiogenic activity.

HCC is a typical hypervascular tumor and angiogenesis accounts for its malignant biological behavior and poor prognosis. Accordingly studies of anti-angiogenic therapy for HCC are critically significant. *In vivo*, the MHCC97 human HCC cell line was employed to establish a highly metastatic nude mouse model of liver orthotopic xenografts to observe the effects of scFv-Ang2 on HCC angiogenesis and tumor growth. The results revealed that the tumor weight and volume in the treated group were statistically lower than those of the control group, indicating that scFv-Ang2 has a significant inhibitory effect on the growth of HCC. The animal model employed in this study has a 100% metastasis rate in the liver and lungs under normal growth conditions and is a common model for intervention in human HCC (47). It was observed in the present study that scFv-Ang2 significantly inhibited the spread of the metastases to the lung and the intrahepatic metastasis of HCC in nude mice (P<0.05), despite the 100% metastasis rate in the liver and lung in both groups. Expression levels of CD31, which is considered as an endothelial-specific marker, were examined using monoclonal CD31 antibody. The results suggest that the expression levels of CD31 in the experimental groups were extremely low, whereas in the control group CD31 was strongly expressed. Accordingly, MVD in the experimental group was much lower than that in the control group (P<0.05). MVD is the most common target reflecting neovascularization. The reduction of MVD in this study suggests that scFv-Ang2 had inhibitory effects on angiogenesis. The process of angiogenesis is a highly complex network of systems. In this study, the angiopoietin pathway was targeted by purifying human scFv-Ang2 and testing its effects on the angiogenesis and tumor growth of HCC in nude mice, which showed that the inhibition of Ang-2 is an important mechanism of anti-angiogenic action.

## Figures and Tables

**Table I:** Screening results of angiopoietin-2 from a phage display antibody library.

Round of panning	Input phage (pfu/ml)	Eluted phage (pfu/ml)
1	4.25×10^13^	3.01×10^5^
2	3.01×10^13^	2.04×10^6^
3	4.04×10^13^	3.68×10^6^

**Table II:** Proliferation and migration of HUVECs in different groups (cultured for 48 h).

					VEGF+Ang-2+scFv		
					
Assay	Control	VEGF	Ang-2	VEGF+Ang-2	1×10^11^ pfu/ml	2×10^11^ pfu/ml	4×10^11^ pfu/ml
MTT	0.7286±0.0347	0.8379±0.0472^a^	0.7469±0.0692	1.1918±0.1973^a^	0.9718±0.0913^b^	0.8396±0.1083^b^	0.7492±0.1314^b^
Migration	21.13±2.02	41.85±9.10^a^	28.74±3.51^a^	61.45±10.08^a^	43.55±4.76^b^	34.56±5.44^b^	29.76±1.35^b^

Data obtained by MTT assay are expressed as mean ± SD of the absorbance at 490 nm. Data obtained from the migration assay are expressed as mean ± SD of cells/scope.

a P<0.05, compared with the control group;

b P<0.05, compared with the VEGF+Ang-2 group.

HUVECs, human umbilical vein endothelial cells; VEGF, vascular endothelial growth factor; Ang-2, angiopoietin-2; SD, standard deviation; scFv, single-chain antibody.
